# Transforming Scientists’ Understanding of Science–Society Relations. Stimulating Double-Loop Learning when Teaching RRI

**DOI:** 10.1007/s11948-020-00208-2

**Published:** 2020-03-16

**Authors:** Maria Bårdsen Hesjedal, Heidrun Åm, Knut H. Sørensen, Roger Strand

**Affiliations:** 1 Department of Interdisciplinary Studies of Culture, Norwegian University of Science and Technology, NO-7491 Trondheim, Norway; 2 Department of Sociology and Political Science, Norwegian University of Science and Technology, NO-7491 Trondheim, Norway; 3 Centre for the Study of the Sciences and the Humanities (SVT), University of Bergen, PB7805, NO-5020 Bergen, Norway

**Keywords:** Science–society relations, Reflection, Responsible research and innovation, Biotechnology, Double-loop learning, Ph.D. training

## Abstract

The problem of developing research and innovation in accordance with society’s general needs and values has received increasing attention in research policy. In the last 7 years, the concept of “Responsible Research and Innovation” (RRI) has gained prominence in this regard, along with the resulting question of how best to integrate awareness about science–society relations into daily practices in research and higher education. In this context, post-graduate training has been seen as a promising entrance point, but tool-kit approaches more frequently have been used. In this paper, we present and analyze an experiment—in the format of a Ph.D. course for early-career researchers—deploying an alternative approach. Drawing on Argyris and Schön’s (1974) framing of reflective practice, and their distinctions between espoused theories and theories-in-use, the analyzed course endeavored to stimulate double-loop learning. Focusing on dislocatory moments, this paper analyses how the course tried to teach participants to reflect upon their own practices, values, and ontologies, and whether this provided them with the resources necessary to reflect on their theories-in-use in their daily practices.

## Introduction: Training for Responsibility

For a long time, research policy communities have given increased attention to science–society relations. At the European level, policies directed at such issues have gone through several shifts. “Responsible Research and Innovation” (RRI) is a recent development, where the underlying idea is to promote a set of practices to help shape research and innovation to respond to society’s general needs and values (von Schomberg 2011). Considering these policy efforts, we may ask how science–society policies, such as RRI, are translated into practice. There are several approaches and strategies to achieve or “do” RRI. However, translating RRI into practice has proven to be anything but straightforward (Macnaghten et al. 2014; Fisher and Rip 2013; Owen et al. 2013; Ribeiro et al. 2016). Existing scholarship on the issue points to substantial challenges in aligning RRI policy with research and innovation practices (Solbu 2018a; Åm 2019a, b; Davies and Horst 2015; Blok and Lemmens 2015; Glerup et al. 2017; van Hove and Wickson 2017).

In order to amend the situation, there has been a range of RRI training initiatives. Many of these are web-based, offering what they often present as tool-kits and with instructions for using these tools.^1^ The number of such tools has grown at great speed. For example, the website www.rri-tools.eu contains links to more than 1100 descriptions of tools and procedures. Post-graduate research training represents another possible strategy for creating and developing RRI awareness, for teaching RRI skills such as public engagement, and for helping early-career researchers in the natural and engineering sciences to engage with science–society relations to a greater extent (Mejlgaard et al. 2018; Bernstein et al. 2017; Tassone et al. 2018; Heras and Ruiz-Mallén 2017; Limson 2018). These efforts also tend to employ tool-kit approaches to introduce researchers to RRI. In this paper, we present and discuss an alternative approach that aims to nurture sensitivity to important underlying aspects of RRI, such as reflexivity and responsiveness with regard to social concerns (Stilgoe et al. 2013). We argue that this alternative approach potentially is a more fruitful way of aligning the intentions behind RRI with research and innovation practices than training in the use of instrumental tools, not the least due to a greater focus on developing reflexive skills.

This effort is in part motivated by deliverables from EU projects that point to “a lack of knowledge about how to develop RRI-curricula in HE curricula and about RRI capabilities” (Tassone et al. 2018, p. 339; cf. McKenna 2016; Mejlgaard et al. 2016). The EU project EnRRIch has worked with higher-education policymakers to map the links between RRI and policy priorities for teaching and learning in higher education. Teachers’ concerns (de Vocht et al. 2017) and adaptions (Okada et al. 2018) to RRI in teaching as well as reports from inclusion-oriented deliberations and actions in emerging RRI practices that focus on scientists (de Jong et al. 2016) have also received attention. Still, it is not clear how one should teach RRI and what the content of the training should be. This knowledge gap becomes even more apparent when we are concerned with the efficacy of RRI training.

This paper addresses this knowledge gap by presenting and analyzing an experimental 5 ECTS Ph.D. course that aimed to prepare early-career biotechnology researchers for engaging with science–society relations by strengthening participants’ ability to reflect on RRI challenges and opportunities. The field of biotechnology comprises many disciplines, including biology, organic chemistry, computational modelling and medicine. This research is very relevant to RRI due to its potential for risk and other, potentially transformative effects on society. Consequently, the field has been given substantial attention in the RRI context. The course ran in Norway during the spring of 2018. Its main underlying idea came from the observation that RRI is based on previous social science and humanities scholarship on emerging technologies (Rip et al. 1995; Nowotny et al. 2001; Irwin 2006; Felt and Wynne 2007; Callon et al. 2009; Felt et al. 2013). This paper highlights the potential benefits of teaching the intellectual background for RRI as a resource for reflecting about science–society relationships, rather than mainly focusing on arguments why RRI is needed and how to use tools for doing RRI in research and innovation projects. In this manner, the paper addresses the wider RRI community as well as scholars engaged in training scientists and engineers in ethical issues and science-technology-society concerns.

## Challenges of Reflecting About Research and Innovation

Our point of departure is that RRI training can learn much from Chris Argyris’ and Donald Schön’s work on teaching professionals about reflection. In particular, their concept of ‘double-loop learning’ may be an appropriate and effective guiding principle for RRI training. This is due to the general emphasis on reflection as an important goal and a key competence both in the European commission’s approach to RRI and in Stilgoe et al.’s (2013) widely cited framework for responsible innovation. The European Commission (EC) emphasizes what it calls six policy keys that RRI should advance: ethics, gender equality, governance, open access, public engagement, and science education. Stilgoe et al. (2013) offer a more scholarly oriented alternative that highlights four dimensions of what it should mean to do RRI: anticipation, reflexivity, inclusion, and responsiveness. This approach also provides a theoretical consistency that facilitates teaching, compared to the topical articulation of RRI of the EC.

The dimensions presented by Stilgoe et al. originate from a set of questions (product, process, and purpose questions) found in public debates about emerging technologies and new areas of science in the UK (ibid., p. 1570). In their RRI framework, *reflexivity* is described as asking “scientists, in public, to blur the boundary between their role responsibilities and wider, moral responsibilities. It therefore demands openness and leadership within cultures of science and innovation” (p. 1571). Stilgoe et al. argue that institutional reflexivity is needed in governance and that reflexivity at this level means holding up “a mirror to one’s own activities, commitments and assumptions being aware of the limits of knowledge and being mindful that a particular framing of an issue may not be universally held”. They call this ‘second-order reflexivity’, referring to Schuurbiers (2011). Stilgoe et al. also propose a set of indicative techniques and approaches to strengthen reflexivity, such as focus groups, consensus conferences, and collaboration with social scientists (Stilgoe et al. 2013, p. 1573).

When teaching RRI, the challenge then is to find effective ways of developing early-career researchers’ reflective competence, which arguably is an important basis for addressing other RRI keys or dimensions. For example, Mejlgaard et al. (2018) find that critical reflection is “of vital importance when teaching RRI or RRI related issues in higher education” (p. 7), and they identify problem-based learning (PBL) (Wood 2003) and inquiry-based learning (IBL) (Hutchings 2006) as two promising teaching methods. Importantly, they recognize that

“the aim of RRI is not that students know the specific concept and terminology of RRI, but that they know how to practice reflexivity: that they can interpret their context, think and act responsibly in research and innovation processes, or in other words, that they possess administrative ability” (Mejlgaard et al. 2018, p. 604).

One may argue that reflection has long been a key idea in education. A classic example is Dewey’s *How We Think* (1933). Since then, more has been written on the features and the importance of reflection, for example Kolb’s (1984) experimental learning theory and cycle, Argyris’ and Schön’s (1974) theory of action/espoused theory/theory-in-use and double-loop learning, and Schön’s work on the reflective practitioner (1983, 1987). These concepts remain central in the field of organizational learning (e.g., Basten and Haaman 2018).

In more recent research on reflection in teaching (Bharuthram 2018; Smith and Trede 2013; Sunderland et al. 2014; Edwards and Thomas 2010), a common denominator is that scholars caution against having an instrumental understanding of reflection. Boud and Walker consider the increased attention to reflection and reflective practice, warning that

“(a)longside these positive initiatives have grown more disturbing developments under the general heading of reflection. They have involved both misconceptions of the nature of reflection which have led to instrumental or rule-following approaches to reflective activities, and the application of reflective strategies in ways which have sought inappropriate levels of disclosure from participants or involved otherwise unethical practices” (Boud and Walker 2006, p. 191).

Moreover, as Beauchamp (2015) notes in her literature review of studies of reflection in teacher education, reflection is a complex concept that should be addressed as such rather than as an educational tool.

The concept of double-loop learning is particularly useful in the RRI context as a strategy to stimulate reflexivity. Argyris and Schön distinguish between single-loop learning, which is learning without changing one’s mental model of the problem at hand, and double-loop learning, which includes a feedback loop that allows individuals’ and organizations’ experience to result in reconsideration and revision of the mental model. In this paper, we use their way of framing reflective practice in organizational learning (Argyris and Schön 1974) as inspiring learning objectives of RRI-related training but also as a point of departure to assess our teaching experiment.

According to Schön (1983), reflective practice is the ability to consider carefully one’s actions so as to be able to engage in continuous learning. Thus, as previously noted, reflective practice is a key competence to engage with the four dimensions of responsible innovation as outlined by Stilgoe et al. To be sure, scientists (like all professionals) can hardly avoid engaging in some form of reflective practice, but—as Schön (1983, p. 243) argues—“they seldom reflect on their reflection-in-action” (p. 243), that is, there is little double-loop learning. Consequently, the art of reflection is under-articulated and thus remains inaccessible to other people than the individual professional who is reflecting introvertly. It was exactly this problem of organizational learning that was a central issue in the scholarship of Schön and Argyris.

An important point of departure of Argyris and Schön’s approach is the distinction between espoused theory and theory-in-use. Espoused theories are those that individuals claim to follow. Theories-in-use are those that can be inferred from studying the individual’s action (Argyris et al. 1990, p. 82). For example, instrumental tool-kit approaches run the risk of producing merely an espoused theory (say, of RRI) among its users rather than a theory-in-use. If so, the result is what Argyris and Schön (1974) call single-loop learning, because the previously existing (non-RRI) mental model of the problem to be managed will remain unchanged and still be the theory-in-use regarding practices of responsibility. In turn, this results in the repeated use of the same approach to the problem of responsibility.

We believe that the overarching ideas and values articulated through RRI policies, such as the dimensions articulated by Stilgoe et al. (2013)—anticipation, reflexivity, inclusion and responsiveness—require double-loop learning, in the sense that scientists should be trained to reflect on their daily practices in the context of RRI as an espoused theory. The training would encourage participants to reflect on the differences and discrepancies between espoused theory and theory-in-use, to question both, and in some cases to reconsider and revise the latter. Thus, we did not embark to develop a course that covered all aspects of RRI.

## Method

The organization of the Ph.D. course that this paper presents, analyses, and discusses, is outlined in the next section. It was inspired by Argyris and Schön’s way of framing organizational learning. However, we did not aim to contribute to the development of their theories or theories regarding the role of reflection in teacher education. As previously noted, Argyris and Schön’s concepts helped us to shape the course, above all by clarifying the benefits of engaging the participants in the intellectual traditions that informed the understanding of science–society relations of RRI policies rather than presenting RRI as an espoused theory. These traditions were explained in depth. It was an implicit assumption that any exploration of science–society relations entails learning, but the presentation of the intellectual background of RRI was meant also to invite the course participants to reconsider their ontologies: “which resemble passing through a portal, from which a new perspective opens up, allowing things formerly not perceived to come into view” (Land et al. 2010: ix). Arguably, double-loop learning stimulates processes that can lead to a shift from one set of preconceptions of the world to another.

Besides, the course was based on the assumption that RRI does not make sense as a source of reflection unless one is familiar with research that shows how development of science and technology is neither pre-determined nor value-free, and unless one understands why engagement with society is called for (Åm 2019b, p. 175). A further conjecture was that the participants, all of whom were recruited from the life sciences, were unlikely to be deeply knowledgeable about such research previous to the course. Rather, we believed that they held beliefs closer to the so-called received view of science (Rommetveit et al. 2013) as a value-free enterprise of producing objective knowledge. The course was intended as a counter-point to this view.

We analyze whether this effort to stimulate double-loop learning was effective, using data generated through participant observation in the course by the first author who wrote extensive field notes. We chose to use participant observation because this allows a documentation of processes and events that cannot be reconstructed with similar validity through retrospective interviews. The second and fourth authors developed and conducted the course but did not collect data during the events. The first author acted as the lecturers’ teaching assistant in preparing group work and in providing feedback on the homework assignment but focused singularly on her fieldwork during the course days. After the course, all the authors carefully analyzed and discussed the field notes and the collected texts. In this, the third author contributed with an important outsider perspective to the analysis, which was conducted as follows.

The first round of working with the data dealt with ordering what happened during the course and plenary dialogues. The second round of analysis examined participants’ articulated reflections and categorized discussions, comments, and reactions to the course topics into different kinds of expressed reflections. In this second round, the analysis focused on moments of disruption or dislocation. This was based on the presumption that early-career researchers first need to recognize that things could be otherwise in order to reflect and question their routine practices in action and to become prepared for disrupting existing orders. ‘Moments of dislocation’ (Howarth 2000, p. 111; Åm 2019a, p. 458) can trigger such recognition.

Dislocatory moments occur when people become aware of discrepancies between their established practices and other practices, views, identities, or organizational policies. Such moments may trigger learning processes that encompass the revision of mental maps, that is, double-loop learning (Schön 1983). For dislocation to happen, RRI-training therefore needs to aim at “ontological transformations that are necessarily occasioned by significant learning” (Land et al. 2010: xi), unhinging early-career researchers’ mental maps about science and society. In the following, we describe how the analyzed course set out to reach this aim.

## Course Organization

As indicated, this paper is an account of a Ph.D. course for early-career biotechnology researchers called “Science, Technology and Society: RRI Course Digital Life Norway”, to share the experiences from organizing and running this initiative. The main objective of the course was to enhance participants’ knowledge of how scientific work is intertwined with changes in society and to introduce them to ideas of democratization of science and current international discussions on RRI. The course introduced the body of academic knowledge that according to our reading of the RRI concept underpins it, namely science and technology studies (STS) and the history, philosophy, and sociology of science. The course combined lectures that presented theoretical insights with long group discussions and hands-on exercises that trained participants how to reflect in the context of their research projects. Deliberation about RRI as a guide to good research practices was also part of the course. The participants were introduced to the definition of RRI in EU’s Horizon 2020 program, the RRI framework as outlined by Stilgoe et al. (2013), how this framework has been integrated into policies articulated by the Research Council of Norway, and how research grant applications could be assessed with a view to the way RRI concerns were addressed in the project plan.

The course was offered through the Centre for Digital Life Norway’s Research School (DLNRS). Digital Life Norway (DLN) is a national center for biotechnology training, research, and innovation. The course consisted of two three-day sessions held two months apart, at two locations in Norway during spring 2018. Recruited mainly from DLN research projects, participants came from four cities in Norway. Seventeen biotechnologists participated throughout the course: thirteen Ph.D. students, three postdoctoral fellows, and one researcher. Except for those participants working on the same project, most participants did not know one another beforehand. The participants’ disciplinary backgrounds varied from molecular systems biology, organic chemistry, and microbiology, to various kinds of computational modelling, medicine, and environmental toxicology, to chemical neuroscience and microbial biotechnology.

The course was structured through envisioned learning goals for each session that built on each other. The topics of the first three-day gathering were, in order, interdisciplinarity, “exploring societal dimensions of science”, “governance and regulation of biotechnology”, “self-regulation and ethical guidelines”, “science and innovation policy”, as well as “Responsible Research and Innovation in theory and practice”. The topics of the second gathering two months later were “co-production of science and society”, “transdisciplinarity”, “public engagement”, “gender and science”, “responsibility conditions”, “risk, uncertainty, post-normal science” as well as “life sciences and modelling”. Thus, the content of the course clearly reflected its ambition of focusing on the understanding of science–society relationships that underpins RRI.

In the following, we focus on dislocatory moments that we identified during this Ph.D. course. When we analyzed the field notes of the first author, some moments in the course stood out as showing a change in the participants’ expressed perceptions, thoughts, or opinions of a topic, which suggested new reflections and responses by the participants that seemed to reflect a shift in their ontologies. We identify tendencies towards double-loop learning and reflection-in-action in these moments.

As mentioned above, the analytical focus on such moments was a conscious choice because we wanted to empirically highlight situations in which we observed instances of double-loop learning and/or initiatives meant to stimulate such learning and thus reflection-in-action. The selected moments are meant both as concrete examples of the perceived efficacy of the methods used to engage early-career researchers in higher education in science–society relations and as potential demonstrations of reflection and double loop learning in practice. To assess whether double-loop learning and reflection-in-action took place, we analyzed the selected moments to see if the participants used input from the course to reflect on their own practices (theories-in-action) and their understanding of these practices. The moments in the subsequent section are listed in the order in which they occurred during the Ph.D. course.

## Dislocatory Moments: Reflection-in-Action

### Moment 1: Governing and Regulating Biotechnology. Learning to Take Pluralist Stances

The envisioned learning goal of the following exercise was learning to take pluralist stances, and the topic of this session was governing and regulating biotechnology. This was during the first gathering. In advance, the participants had been assigned a set of readings (Baltimore et al. 2015; Jasanoff et al. 2015; Sarewitz 2015; Biotechnology Advisory Board 2018) and directed to watch on YouTube a video debate titled “CRISPR: To eat or not to eat. Debatt om genredigert mat [A debate on gene-edited food]” (https://www.youtube.com/watch?v=x2hKhSJ9qEM). In light of ongoing revisions of the Norwegian Gene Technology Act, we asked them to consider the following question during their preparation for the course: “What is the public invited to comment upon?” Regarding the CRISPR debate, we assigned participants the following task: “Map the arguments in this debate. What are the representatives of civil society concerned about? What are the counter arguments?”

The group exercise resulted in a panel debate in one classroom. The participants were divided into three groups. Each group was assigned one of the following roles: researchers; a network of GMO-free food and Greenpeace activists; and representatives of the relevant industry. The participants were asked to analyze the provided material (public comments from these actor groups derived from media debate and web research conducted by teaching assistants in advance) and then to prepare arguments and statements for a public debate on CRISPR. Each of the three groups should select two representatives to participate in the panel debate (six participants). The remaining 11 participants and the lecturers comprised the audience; after the panel presentation, the audience asked questions of the six panel participants.

The participants valiantly endeavored to convince the others that their assigned view was the best one. Even though some of the participants enthusiastically embraced their assigned roles from the beginning, exaggerating the assumed views, the longer the debate went on, the more the participants seemed genuinely to try to argue their designated points of view. After the debate was over, one of the participants who had been placed in the Researcher group defending the CRISPR technology told the class that “I have to clarify that my position in this panel is not my personal point of view, and I [personally] don’t agree with what I just said [in the debate]. It pained me to defend this point of view.” Some of the others also reported that they had found it hard to defend the position they had been assigned.

Focusing on taking pluralist stances contributed to starting the process of reflection on science–society relations amongst the participants. Having to spend time to familiarize and then defend someone else’s point of view is a frequently used technique. It was interesting to see how most of the participants became so engaged in the discussion defending someone else’s view that they afterwards felt the need to clarify that this was not their personal opinion.

### Moment 2: Engaging in the Broader Context of Your Work: Meeting with the Director of Digital Life Norway

The second dislocatory moment came during a meeting with the director of the national Digital Life Norway (DLN) center. Before he arrived, there was a session on the topic “Science and policy: where does the money come from and why do they come?” The session on regulation and funding highlighted the co-production of science and society. In particular, the session aimed at increasing participants’ understanding that research is part of a larger context and intertwined with other stakeholders’ goals. The aim of the session was to help participants grasp the importance ascribed to science and innovation in Norwegian politics today, and to increase their understanding of the mandate Norwegian policymakers give to research actors. It was considered important that early-career researchers are aware of what society wants from funding research. After a lecture based on Gibbons’ (1999) ‘new social contract’ and Vermeulen’s (2009) work on ‘making big science’, the director of the DLN held a presentation about the center. Beforehand, participants were required to read the RCN’s research policy document underlying the establishment of the DLN and to prepare questions for the visitor.

The first question came only a few minutes into the director’s talk. The participants asked the visitor many important and challenging questions that were critical to DLN, and they were deeply engaged in the discussion. In this example, we traced double-loop learning from the kind of questions the participants asked as well as from the context and reflections they offered in explaining their questions. Questions included, for example, who the stakeholders were that DLN was supposed to engage with; what “Digital Life” really signified; what was meant by ‘transdisciplinarity’ in the policy document and how this was supposed to be achieved; and how inclusive the center should be in terms of accommodating new biotechnology projects.

We interpret this as evidence of double-loop learning because we observed that the participants asked questions that prompted the director to reflect on his espoused theories. These happened to resemble the espoused theories, which the students discovered that they used themselves at the beginning of the course. Through these questions, the participants raised important issues regarding the wider implications of Norwegian biotechnology, applying course literature, and adding a higher level of reflection than we previously had seen in the course.

### Moment 3: ELSA Issues. Getting Others’ Views on Your Project

The third dislocatory moment happened during a group exercise on Ethical, Legal and Social Issues or Aspects (ELSI/ELSA). As a preliminary step to the exercise, the participants listened to a lecture that aimed to provide an understanding of where RRI was coming from, such as the previous policy program of ELSI/ELSA. The lecturer argued that an activity is not necessarily responsible just because there has been engagement with risk assessment, ethical issues, or technology assessment. ‘Responsibility’ should signify wider concerns.

After this lecture, the participants were divided into four groups to discuss the projects they were working with, in separate rooms. First, the participants were to take three minutes to explain their project to the others in their group, and then the group was to spend five minutes identifying and discussing possible issues of concern in the project before moving on to the next. The point of the exercise was to utilize and underscore the fact that the participants were publics to each other’s specialized projects. During this discussion, and unlike previous exercises, all the groups closed the doors to the rooms they sat in. We interpreted this as a sign that they wished to discuss these matters in peace and quiet, without anyone else listening.

After the exercise, the groups reunited and presented a short plenary summary of their discussions. Interestingly, we observed that several participants were surprised about how the others in their group regarded their project. One participant commented that “I never knew there were so many problematic aspects of my project!” At the end of the course, one participant commented that this exercise had been the most significant group activity because she received crucial input with perspectives on her project that she had not considered before. Such experiences indicate an increased awareness and understanding that the public may interpret a project significantly different from the participating scientists. Thus, the lesson was that the task of identifying concerns should not be done only by those undertaking the research. Such engagement should also involve actors with other interests and points of view.

### Moment 4: Discussing “Something” Learnt

At the beginning of the second gathering of the course, after a break of two months, the participants were asked how they experienced the aftermath of the first gathering. Several participants described feeling that they had learnt something important. The challenge was to articulate what this ‘something’ was. They said that they had been unable to describe to their colleagues at home what they actually had learnt during the first gathering. One of the participants said that “I have tried to explain to people what I’ve been doing for three days. It’s been difficult. I want to go back to the lunch discussions [at the department] and quietly ask some questions [about how and why we do things].”

The problem of finding the right words to describe what was achieved by taking the course re-occurred in the reflection notes that the participants wrote on the final day. One of them wrote that “It is difficult to put into words the learning outcome. I feel that I have learnt a lot about RRI, and this has changed and influenced my mindset regarding my view of science. But it is difficult to put into words.” Another participant expressed that “After the course, I still feel RRI is hard to grasp, but a lot of new thoughts have come up. When we have discussed the theories, I follow [along], and have also been able to participate in some discussions. But I don’t think I can explain this [RRI] very well to others”. The challenge involved in learning how to communicate in an interdisciplinary manner what was important lessons from the course was noticeable and probably also constituted a useful experience for the participants. Developing a shared language for thoughts and reflection is essential for good discussions. We provided the participants with some relevant concepts such as co-production, technological determinism, technological fix, and value pluralism, but we are not sure that participants integrated them in their vocabulary by the end of the course.

### Moment 5: Homework

At the end of the first three-day gathering, the participants were given a two-part homework assignment. First, all seventeen participants were asked to draw a map of relations identifying the actors, ideological context (promises, visions), conceptual frameworks, disciplines, funding institutions, regulations, instruments, and other relevant aspect of their Ph.D./research project. In the second part of the homework, the participants could choose one of six assignments. Five chose to send comments to the public hearing on the proposed revisions of the Genetic Engineering Act; one wrote a newspaper commentary on her research; one analyzed the gender balance in her research environment, one group working on the same project planned a RRI workshop; and two participants sought input on transdisciplinary workshop tools that they tried to implement in their project.

On the first day of the second gathering, the participants presented their homework and their reflections. This day, the lecturers’ response method was to give input on relevant topics as they emerged from the presentations. Based on a review of the homework, lecturers had prepared concise input on the following, emerging themes and concepts: co-production, transdisciplinarity, public engagement (who is the public?), and science communication. From the maps of relations in the participants’ presentations it was clear that many of the participants struggled with considering the broader context of their project. Most of the participants focused on human actors and gave less priority and attention to the contexts that influenced their projects, such as value systems, widespread beliefs or ideologies, funding and regulations. However, some of the participants excelled in this task. They had done a solid effort and presented complex maps that included both human and non-human actors, including political, ideological, and environmental contexts.

We consider the homework presentations important because they, along with the review of the relational maps, led to a discussion of what a relevant context might be. Several participants commented that they had not thought that ideology, values, underlying principles, and their discipline should be indicated on their map. This event challenged each participant to critically review her or his own map while considering the maps of the other participants and reflecting upon what could be improved. This event was also an example of how to teach another way of thinking, involving other aspects and actors outside of what is often seen as the “purely scientific” aspects of a research project.

### Moment 6: Imagining Desirable Futures

The sixth dislocatory moment occurred during a group exercise about imagining desirable futures. As a starting point for the exercise, the participants heard a lecture about responsibility conditions, followed by an extensive plenary discussion about transparency in science. Then they were introduced to the concepts of risk, uncertainty, and post-normal science as a background to learn about the concepts ‘risk society’ and ‘reflexive modernization’. The participants were split into four groups and asked to discuss and propose first probable and then desirable futures. “How should the future (of science) look in 30 years?” After the group exercise, all groups presented in a plenary their desirable futures. Most of the groups used as their starting point what the world looked like 30 years ago. In the ensuing exchange, it became evident that the groups had struggled with proposing desirable futures. Rather, they tended to discuss only *probable* futures. A few participants also expressed thoughts such as “there is no point in discussing this because we don’t know what will happen anyway.”

During the group and plenary discussions, we observed that the participants struggled to negotiate the meaning of the desirable versus the probable. The following exchange is from one of the groups where two participants were discussing what role science should play in a desirable future. One of them asked “Are we talking about probable or possible futures? When you say that we are more tied to devices—do we wish this?”. The second participant answered “Yes!” To this the first participant replied “Oh! That’s not desirable for me”. Another example of the way probable and desirable outcomes were negotiated was the following exchange about the future organization of a desirable research system. One participant argued that “I think it will be more interdisciplinary” while another objected that “This is about desirable futures”.

The lesson was that what some find desirable may not be desirable to others. On an intellectual level, this was not a new insight to the participants, which they demonstrated in the discussions before this group exercise. However, experiencing in practice that they did not agree with one another about the features of a desirable future for research policy and the university system seemed to surprise the participants a great deal. Thus, we observed that it stimulated their reflection-in-action and their double-loop learning in practice. Apparently, the participants improved their understanding of how values and priorities influence political as well as scientific decisions, recognizing that the future is not pre-determined but a result of conscious choices. Hopefully, this helped some of the participants to adjust their priorities and their normative ideas about what science should be.

### Moment 7: Discussing the Use of Models in Science

The seventh and final important development took place during the final day of the course. It exemplifies how the course challenged the participants’ perspectives in order to foster double-loop learning, in this case through a session on the use of models in science. This session involved one individual exercise and one group exercise, followed by an extensive plenary discussion about the use of models. The goal was to raise issues with the traditional reductionist understanding of models; in particular by showing how representation is closely connected to intervention. This was meant as an invitation to reflect about how science does and does not affect society. The discussion revolved around what models scientists use as well as what models can teach their users.

The individual task asked participants to describe the biological question that their projects wanted to answer and their models for researching this. After a discussion about these issues, they were challenged to come up with reasons why the models they use in their project (1) do not represent reality, (2) will almost certainly be irrelevant, and (3) tell little about the questions that they are asking in their projects. Through these provocative statements, they were invited to reflect about potential weaknesses of their models and how such problems might affect their research.

The field notes from the discussion show that—although highlighting what they saw as a necessary reduction of complexity in models—quite a few of the participants did not think about models as intervening in but as representing reality. Thus, they assumed that they would be able to know how their problem-world worked when all parameters and variables were considered. A participating microbiologist, reacting to a comment by one of the lecturers that we cannot know all the parts of the world separately and then put them together, said that “this is just limited by our capacities, such as data power, storage and time. This will evolve.”

During the discussion, however, other reflections emerged. One participant commented about his own work that “The sample sizes are too small. And we use predefined assumptions”. Another participant, also relating the topic to his own work, argued that “Stem cells are too simplistic. What would happen if we used a dead body [other than the one we are using], not a man 42 years old? We think that the brain structures are similar [across bodies], but this is not always the case.” This was followed up by a participant’s more general comment on models.

“My first point is: It’s just models. It’s the current view of biotechnology. In the models in my project, we assume a stable state. But there are more than three hormones in [the animal we study]. They don’t only have one temperature. These are difficult things to test in an experiment. Therefore, we are guessing on parameters. Nature is complex”.

Toward the end of the discussion, another participant also commented on the difficulty of managing complexity. “Our models are simplistic. The liquid composition is not exactly as it is—it is fundamentally flawed.”

These quotes represent only some of the participants’ reflections on their use of models and on what models may and may not tell us. They exemplify double-loop learning through the emerging reconsiderations of their ontologies or their epistemological assumptions as the participants challenged their own models and saw them in a broader context. From this perspective, they assessed what models actually can tell about the relevant physical world.

The quotes also show how the participants’ perspectives changed and their willingness to critically discuss and assess the use of models in science. This does not mean that everyone agreed with a critical perspective on models. However, after the discussion, several participants reflected upon these different perspectives, saying that for them, this was a new way of thinking about the use of models in science. Thus, the seventh dislocatory moment demonstrates the importance of creating arenas for discussion of and reflection on topics that often are not addressed in everyday research practices. In turn, as we saw, such situations invite consideration regarding participants’ scientific practices, their theory-in-action, rendering change possible.

## Discussion: Assessing Double-Loop Learning Efforts

Biotechnology is a heterogeneous field of researchers from many, often neighboring disciplines. This was reflected in the participation in the course, and the discussions and some of the group work showed that developing an interdisciplinary understanding between presumably close fields can be both challenging and beneficial. Given that people tend not to see what is surprising in practices that they consider “the normal way of doing things”, engaging with others as they did during the course helped the participants to see “what [she/he] ha[s] worked to avoid seeing” (Schön 1983, p. 283). Thus, the interdisciplinary situation of the course and the plentiful opportunities of the participants to be the others’ publics potentially introduced fault lines (Traweek 2000) in the discussions that took place. These fault lines were helpful in facilitating double-loop learning.

The course goal was for participants to be able to engage in broader debates surrounding their research, to address social, ethical, political, and economic aspects of their work, and to critically reflect on the R&D system and to take part in initiatives for its improvement. Thus, reflection played a crucial role in the course with the intention of facilitating double-loop learning. This was the underlying curriculum of the sessions. Moreover, for many of the participants, this was the first time they learnt about biotechnology from a social science point of view, which introduced an additional interdisciplinary fault line. This challenged participants to consider their scientific practices in new ways. The discussion about models mentioned earlier was an example of this. This effect was also present in participants’ accounts of their benefits from the course, which supports the assumption that double-loop learning happened throughout the course.

In the dislocatory moments noted above, participants had to reflect upon their own practices, values, and ontologies. This helped them develop a meta-perspective on the conduct of such reflections. Though we identified instances of double-loop learning in each of the seven dislocatory moments, it was their sum, rather than each moment in itself, that provided double-loop learning and reflection-in-action to facilitate engagement in RRI. The first moment initiated a process of reflection on the implication of one kind of emerging biotechnology, establishing a starting point for asking critical questions about the relation between Norwegian biotechnology and the wider society and its stakeholders outside of academia.

This process continued in the meeting with the DLN director. Here, participants had the opportunity to ask critical questions regarding the center’s role, priorities, funding, aims, and the methods for achieving these. The hands-on exercises challenged participants to articulate the wider (and to the participants) more blurred relationships, but they were close enough to the participants’ own projects that they could see the implications for their daily work. Which exercises the participants found most thought provoking or important varied.

If we taught the course again, we would adapt a couple of readings for the first sessions to improve the first group exercise. However, in general, based on the experiences presented in this paper, we do not see a need for substantial changes in the set-up of the course. Dislocatory moment six—the discussion about desirable and probable futures—was also a kind of dislocatory moment for the organizers because we were surprised that so few students shared our assumptions about structural problems with responsibility conditions in today’s academia. We learnt that we should aim to present students with alternative, contrasting points of views in order to trigger critical reflection on the subject. In addition, maybe our evaluation of the situation was too negative.

Admittedly, it is difficult to assess the learning outcome of such a course. First, as we did not interview the participants before the course began, we do not know to what extent their reflections during the course actually emanated from the course or from reflective practices already in place before the course. Second, to assess outcomes properly, we should have followed participants over time to observe if there were long-term changes in their research practices and their view of science. Nevertheless, we claim some effects of the course, based on participants’ accounts of their perceived outcome, because their feedback was overwhelmingly positive. For example, one participant reported that she had gained a new perspective in which science was something also influenced by social actors. “One of the things I take home from the course […] I think I had this vision of science as free and curious, but I’ve been enlightened [by learning about other perspectives]. Now I see how much it’s about governing and ‘catching’ words.”

Another participant emphasized that she had gained new knowledge about science but complained that she found the new insights difficult to apply in her daily work. “This [the course] has really changed my way of thinking, and my ideas about science and my world. But how to do it in my daily work? But it will change how I speak about science, how I present it”. Thus, she interestingly articulated a possible tension between espoused theory acquired during the course and theory-in-use related to her everyday practice, a recognition that could be a first step in a double-loop learning process. The participants also acknowledged that they had learnt something new about citizenship. As one of them formulated it, “I realize [now] that I’m also a citizen. This [course] has changed my way of thinking about the public. Now I think we are all citizens”. It is these two participant accounts that most clearly articulated the kind of ontological transformations that the course was aiming at.

## Conclusion

Policy demands for doing RRI in biotechnology emanate from a belief in the necessity of changing scientific practices and an assumption that this may be achieved by practicing RRI in the context of research projects. However, such achievements are demanding, not least because there is no straight-forward way to translate RRI concerns into competence, going from espoused theory to theory-in-use. The popular idea that RRI can be disseminated and implemented in the form of tools and toolkits risks catering mainly to single-loop learning, due to its emphasis on the implementation of procedures. According to Argyris and Schön (1974), such learning is not effective in providing for change.

The course that we have described and analyzed in this paper was an experiment in teaching RRI, stressing the underlying ideas and the development of reflective competence, following the tenets of double-loop learning. Since the course has run only once, with a limited number of participants, we have to be careful about generalizing the experiences. However, given what happened through the seven dislocatory moments and the feedback from the participants, we suggest that there is considerable potential gain from educating early-career researchers by providing them with the resources to reflect on their theories-in-use of their daily practices. Facilitating double-loop learning in this manner seems important, given that the objective of RRI policies is to produce real change.

In the actual world of research, innovation, and higher education, RRI is but one of many policy principles, and a quite weak one at that. Implementing RRI into early-career researchers’ mental models and not merely into their repertoire of espoused theory seems desirable from the point of view of RRI policies. However, one potential challenge is with the dominant policy narratives of innovation for economic growth, where RRI may be considered a detour. In a review of a broader set of efforts to teach critical reflection on science and technology, it was observed that such efforts indeed may be marginalized because of their success: “constantly under threat ‘every time there is a new dean” (Mejlgaard et al. 2016, p. 19). Still, during the conduct of our course, participants were not concerned that its content could be a problem to potential transitions from research to innovation.

Our course was driven by the desire to move beyond ritualistic reproduction of espoused theory. Mejlgaard et al. (2018) tried to capture this quality by advocating RRI as *phronesis*, that is, as practical wisdom, as opposed to the *episteme* of knowing RRI policies. Our contribution is to propose an approach to obtain that desired change, namely through the facilitation, stimulation, and support of double-loop learning through careful selection of course content and form. Whether the participants actually are able to use the espoused theory of the course to change their theories-in-use in order to reform their daily practices, remains to be seen.

New experiments with more participants, as well as subsequent research on these teaching experiments, are needed. We believe that they will provide additional insights about how to internalize theories-in-use in the context of reflecting about science–society relations. We are however confident that we observed authentic engagement during the course. Moreover, we interpret some of the discussions during the course to indicate that the participants gained knowledge of matters of concern related to biotechnology (Latour 2004, 2008), or what Solbu (2018b) calls epi-knowing. In turn, this should result in an increased awareness of the importance of reflecting about science–society relations.

## Epilogue

About a year after the course, the first author by chance met seven of the participants on different occasions and took the opportunity to conduct spontaneous interviews (Henriksen and Tøndel 2017) with them: Had the course in any way influenced them and their daily work? The general response was that they had learnt a lot and were glad to have taken the course. They said that they would take it again, although they found the lessons challenging to apply in their daily research practices. Their assessment of the effectiveness of the course varied. One participant commented that she felt that she did not learn much scientifically from the course and that it had no effect on her daily work. Other participants reported that the course had influenced them considerably. One of them said that “[s]he [one of the lecturers] opened some doors for me that definitely were not open before [regarding perspectives on science and society].” Another participant added that the RRI course had impacted her and that it “so often and in so many situations pops into my head. I think about it almost every day”. Yet another participant told that during the last year he had repeatedly contacted the research school of DLN to request similar courses or activities, and to find out if the course would be held again.

## Footnotes

1 E.g., EU projects HEIRRI (http://heirri.eu/), Fit4RRI (https://fit4rri.eu/), RRI Tools (https://www.rri-tools.eu/), IRRESISTIBLE (http://www.irresistible-project.eu/index.php/en/), and FOSTER (https://www.fosteropenscience.eu/). For other examples, see: https://www.parrise.eu/other-rri-projects/.
